# Dynamics of driven dissipative temporal solitons in an intracavity phase trap

**DOI:** 10.1038/s41377-025-02147-8

**Published:** 2026-02-18

**Authors:** Nicolas Englebert, Corentin Simon, Carlos Mas Arabí, François Leo, Simon-Pierre Gorza

**Affiliations:** 1 Service OPERA-Photonics, Université libre de Bruxelles (U.L.B.), 50 Avenue F. D. Roosevelt, CP 194/5, B-1050 Brussels, Belgium; 2 Department of Electrical Engineering, California Institute of Technology, Pasadena, CA 91125 USA; 3 Institut Universitari de Matemática Pura i Aplicada, Universitat Politècnica de València, 46022 València, Spain

**Keywords:** Frequency combs, Nonlinear optics, Solitons

## Abstract

Temporal cavity solitons are ultrashort optical pulses circulating in driven Kerr resonators. Their intrinsic stability and ability to generate coherent broadband frequency combs have led to breakthroughs in fields such as sensing, metrology, and signal synthesis. However, this robustness limits control over soliton dynamics and constrains comb characteristics. Here, we demonstrate that stationary and moving trapping potentials, generated through intracavity phase modulation, provide unprecedented control over cavity soliton properties. We theoretically show that, for deep potentials, the soliton spectral shift and repetition rate tuning range are primarily limited by a Hopf bifurcation, and reveal the role of dissipation in soliton dynamics. Using a fibre resonator, we observe stable blue- and red-shifted solitons up to 0.4 times their spectral width, at least an order of magnitude larger than with external phase modulation of the drive. We also investigate the interplay between the trapping potential and stimulated Raman scattering, showing that Raman self-frequency shift can be fully compensated, extending the existence range of cavity solitons. Our results provide a new means for stabilising or rapidly tuning the repetition rate of Kerr combs over a wide range, broadening the applications of Kerr frequency combs.

## Introduction

Dissipative solitons are a fascinating class of localised structures that emerge in non-conservative systems^1–4^. They are uniquely determined by the system parameters to achieve a double balance: between dissipation and gain, and between dispersion and nonlinearity^5^. The latter ensures the invariance of nonlinear waves during propagation, endowing them with particle-like behaviour. Among optical dissipative solitons, temporal cavity solitons (CSs) are sech-type pulses propagating endlessly in coherently driven Kerr resonators^6–9^. The remarkable stability of the emitted output pulse train has led to growing interest in CSs over the last decade, driving progress in diverse applications such as distance measurement^10^, high-resolution spectroscopy^11^, telecommunications^12^, and astronomy^13^. Meanwhile, considerable efforts have been devoted to controlling the properties of cavity solitons^11,14–16^, notably through modulations of the driving field’s phase^17–20^ and amplitude^21–24^. An appealing alternative approach would be to leverage the interaction between solitons and an intracavity phase modulation (IPM), which acts as an external potential for the nonlinear waves^25–28^. The interplay between solitons and a potential was extensively studied for conservative solitons (see e.g., refs. ^29–31^ and references therein), as well as for dissipative solitons in active mode-locked lasers^32,33^. Yet, the frequency constraint and phase locking of CSs to the coherent driving wave make these solitons more susceptible to perturbations affecting the delicate double balance. This raises the question of the effectiveness of intracavity phase traps in controlling and manipulating cavity solitons.

In our work, we theoretically and experimentally explore the interaction between coherently driven cavity solitons and trapping potentials. Specifically, we derive the properties and existence range of cavity solitons synchronised to drifting IPMs. We demonstrate that the trapping enables the robust manipulation of the CS centre frequency and the output soliton comb repetition rate over a larger tuning range than established methods. As an application of the CS frequency control, we study the competition between an IPM and the stimulated Raman scattering (SRS). We show that SRS can be fully compensated and that the trapping mechanism extends the existence of cavity solitons beyond the fundamental limit imposed by SRS alone^34^. Our work, by combining coherent driving and intracavity electro-optics modulation, paves the way to new generations of versatile Kerr comb sources.

## Results

### Theoretical analysis

We first theoretically analyse the impact of a real potential (*V*), such as that generated by IPM, on the existence and stability of CSs. The starting point is the driven-dissipative nonlinear Schrödinger equation, also known as the Lugiato-Levefer equation (LLE), a mean-field model that describes remarkably well the dynamics of coherently driven passive Kerr resonators^35–37^. It is here generalised to include a potential term. As such, it can be seen as the driven-dissipative Gross-Pitaevskii equation. It reads in dimensionless form (see Supplementary Information section A for the normalisation)^28,38^:

1 $$i\frac{\partial A(t,\tau )}{\partial t}={iS}+\left(\left[\Delta +V\left(\tau \right)\right]-i-{\left|A\right|}^{2}+i\left[d\frac{\partial }{\partial \tau }+i\frac{{\partial }^{2}}{\partial {\tau }^{2}}\right]\right)A$$

where *t* is the (slow) time associated with the round-trip evolution of the electric field envelope $A(t,\tau )$ of a wave propagating in a dispersive resonator with anomalous group-velocity dispersion and focusing Kerr nonlinearity. *τ* is a (fast) time variable defined in a co-moving reference frame in which $V(\tau )$ is assumed stationary with time *t*. *S* is the driving amplitude and Δ is the normalised phase detuning from the closest resonance. *d* is a drift coefficient that accounts for a non-zero group velocity at the driving frequency in the co-moving reference frame.

**Fig. 1:**
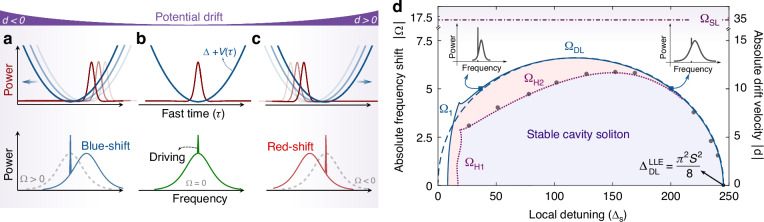
Control of CSs by a potential: concept and bifurcation structure. Trapping of a CS in a potential $V(\tau )$ for negative (**a**), zero (**b**) and positive (**c**) drift *d*. Without drift ($d=0$), cavity solitons are trapped at the potential minimum. Stationary solitons are either blue- or red-shifted, depending on the sign of the drift. **d** Phase diagram in the $({\Delta }_{{\rm{s}}},|\Omega |)$-parameter space showing the region of existence of cavity solitons and their stability for a cosine potential ($J=68$, $W=0.52$), and $S=14$. The horizontal dash-dotted line (${\Omega }_{{\rm{SL}}}$) is the limit set by the maximum slope of *V*. The dashed blue line is the driving depletion limit of CS (${\Omega }_{{\rm{DL}}}$) given by the reduced model Eq. (8), while the two plain blue lines are the saddle-node bifurcations (${\Omega }_{1}$ and ${\Omega }_{{\rm{DL}}}$) of CS solutions of Eq. (1). The Hopf bifurcations (${\Omega }_{{\rm{H}}1,2}$) at which CSs gain or lose their stability are represented by the purple dotted lines. The grey dots show the frequency shift where the CS ceases to exist in direct numerical simulations of Eq. (1) when slowly increasing the drift *d*. The blue and red shaded areas indicate regions of stable and unstable CSs, respectively

Dissipative Kerr cavity solitons are stationary solutions localised along the fast time *τ*. They are sustained by the coherent driving and, without the potential, their properties are uniquely defined by the detuning and the driving strength. Provided that the potential slowly varies on the soliton envelope, it can be linearised around the soliton position ${\tau }_{{\rm{s}}}$, $V\approx V\left({\tau }_{{\rm{s}}}\right)+{\partial }_{\tau }V{|}_{{\tau }_{{\rm{s}}}}(\tau -{\tau }_{{\rm{s}}})$. Equation (1) shows that the potential modifies the detuning seen by the soliton according to its position. We define this detuning as the *local detuning* $\Delta +V({\tau }_{{\rm{s}}})$. Besides, *V* adds a linear phase $\propto {\partial }_{\tau }V$, hence shifting the frequency of the CS. Therefore, stationary CSs in the reference frame of *V* can be understood as follows. A negative (positive) slope at the soliton location ${\tau }_{{\rm{s}}}$ makes it travel slower (faster) than the driving frequency due to anomalous dispersion, stabilising the CS at the potential minimum for $d=0$. Consequently, without drift, the CS spectrum is centred on the driving frequency (see Fig. 1b). Conversely, for $d\,\ne\, 0$, we expect the CS to settle at a position ${\tau }_{{\rm{s}}}$, away from the minimum where it undergoes a frequency shift (Fig. 1a, c), as demonstrated in ref. ^26^ for a very small soliton frequency shift. Yet, little is known about the existence and stability of these solitons for large drifts and frequency shifts. Numerical simulations of Eq. (1) confirm that stable solitons exist up to a critical drift value.

To gain more insights into the properties of trapped CSs, we derive a reduced model by applying the Lagrangian perturbative approach to the solitary wave solutions of Eq. (1)^26,39,40^. Neglecting the background on which the solitons sit, the solution is approximated by a sech-shaped pulse whose centre frequency is shifted by $\Omega :{A}_{{\rm{s}}}\left(t,{\tau }\right)=B\,{\mathrm{sech}}\left[\frac{B\left({\tau }-{{\tau }}_{{\rm{s}}}\right)}{\sqrt{2}}\right]\exp (i[{\phi }-\Omega ({\tau }-{{\tau }}_{{\rm{s}}})])$, where *B* is the soliton amplitude, *ϕ* its phase and ${\tau }_{{\rm{s}}}$ its position along the fast time. This yields four coupled equations, one for each soliton parameter (see also Supplementary Information):

2 $$\frac{{\rm{d}}\Omega }{{\rm{d}}t}=-\frac{\Omega }{B}\frac{{\rm{d}}B}{{\rm{d}}t}-2\Omega +\frac{{\rm{d}}V\left({{\tau }}_{{\rm{s}}}\right)}{{\rm{d}}{{\tau }}_{{\rm{s}}}}$$

3 $$\frac{{\rm{d}}B}{{\rm{d}}t}=-2B+{\pi }S\cos \left({\phi }\right){\mathrm{sech}}\left(\frac{\Omega \pi }{\sqrt{2}B}\right)$$

4 $$\frac{{\rm{d}}{\phi }}{{\rm{d}}t}=\frac{{B}^{2}}{2}-{\Omega }^{2}-\left[\Delta +V\left({{\tau }}_{s}\right)\right]-\left(\frac{{\rm{d}}{{\tau }}_{{\rm{s}}}}{{\rm{d}}t}+d\right)\Omega$$

5 $$\frac{{\rm{d}}{{\tau }}_{{\rm{s}}}}{{\rm{d}}t}=-2\Omega -d$$

We here focus on steady-state solutions. First we find that $\Omega =-d/2$, or in dimensional units ${\beta }_{2}{L}_{{\rm{c}}}\delta \omega =\bar{d}{t}_{{\rm{R}}}$, where ${L}_{{\rm{c}}}$ is the resonator length, ${t}_{R}$ is the roundtrip time, ${\beta }_{2}$ is the group-velocity dispersion coefficient, $\bar{d}$ is the dimensional drift coefficient and *δω* is the soliton frequency shift. The above condition ensures that the soliton remains stationary in the potential, implying that the CS centre frequency is determined exclusively by the drift. Defining locally the gradient of *V* as a monotonic function $D={\rm{d}}V(\tau )/{\rm{d}}\tau$, the CS steady-state temporal position is ${\tau }_{{\rm{s}}}={D}^{-1}{|}_{-d}$, with ${D}^{-1}$ the reciprocal function of *D*. Besides, we have $\Omega =\frac{1}{2}D$. The frequency shift is thus bounded by the maximum value of the slope ${\Omega }_{{\rm{SL}}}=\frac{1}{2}|{D}_{\max }|$. In dimensional units, it reads:

6 $$\delta {\omega }_{{\rm{SL}}}=-\frac{{\mathcal{F}}}{2\pi }\times {\phi }_{\mathrm{int}}^{{\prime} }$$

where ${\mathcal{F}}$ is the cavity finesse and *'* stands for the first derivative with respect to the fast time *τ* of the internal phase modulation. It is worth comparing this result with the limit $\delta {\omega }_{{\rm{SL}}}=-{\phi }_{{\rm{ext}}}^{{\prime} }$ for external phase modulations of the driving^17^. It shows that the maximum frequency shift a soliton can sustain is enhanced by the factor ${\mathcal{F}}/2\pi$ when the resonator is internally modulated. Likewise, the CS speed in response to the potential is enhanced by the same factor, making internal modulation more effective in manipulating CSs (see Supplementary Information, Section E). Moreover, for static potentials ($d=0\Rightarrow \Omega =0$), we see that CSs are located at the extrema of *V*. However, only the minima are stable. Hence, CSs are trapped at the minimum of the potential. The equation of motion for the soliton phase yields the stationary soliton amplitude (or, equivalently, its spectral width $\approx 0.8{B}$):

7 $$B=\sqrt{2[\Delta +V\left({\tau }_{{\rm{s}}}\right)-{\Omega }^{2}]}=\sqrt{2{\Delta }_{{\rm{eff}}}}$$

where we have introduced the effective detuning ${\Delta }_{{\rm{eff}}}$ that accounts for the local detuning ${\Delta }_{s}=\Delta +V({\tau }_{{\rm{s}}})$ and the additional detuning from the frequency shift due to dispersion. The usual expression for the CS amplitude $B=\sqrt{2\Delta }$ is retrieved for $\Delta ={\Delta }_{{\rm{eff}}}$^37^.

Finally, setting $\cos \left(\phi \right)=1$ in Eq. (3) yields the CS existence limit. It reads:

8 $$\frac{2}{\pi }\sqrt{2{\Delta }_{{\rm{eff}},{\rm{DL}}}}=S\,{\mathrm{sech}}\left(\frac{\pi {\Omega }_{{\rm{DL}}}}{2\sqrt{{\Delta }_{{\rm{eff}},{\rm{DL}}}}}\right)$$

The maximum detuning (${\Delta }_{{\rm{eff}},{\rm{DL}}})$ can be seen as the pump depletion limit and corresponds to a saddle-node bifurcation. The predicted value by the LLE ${[\Delta }_{{\rm{DL}}}^{{\rm{LLE}}}={\left(\pi S\right)}^{2}/8]$ is here generalised to frequency-shifted cavity solitons. Specifically, it shows that the driving strength *S* is effectively diminished by the normalised spectral amplitude of the soliton at the driving frequency: $S\to S\,\mathrm{sech}\left(\frac{{\pi }\Omega }{2\sqrt{{\Delta }_{{\rm{eff}}}}}\right)$.

We now particularise to a periodic potential of the form $V\left(\tau \right)=-J\cos \left(W\tau \right)$ where we set the amplitude and frequency to $J=68$ and $W=0.52$, close to the experimental parameters. The existence region of cavity solitons, plotted in the two-dimensional (${\Delta }_{s},|\Omega |$)-parameter space, is reported in Fig. 1. The limitation from the maximum slope is $|{d}_{{\rm{SL}}}|=2|{\Omega }_{{\rm{SL}}}|$. With our parameters, ${\Omega }_{{\rm{SL}}}$ cannot be reached because the depletion limit (${\Omega }_{{\rm{DL}}}$) is met first. Furthermore, the largest frequency shift, determined by $\left|{\Omega }_{{\rm{DL}}}\right|$, shows a maximum with ${\Delta }_{s}$. On the one hand, at large detunings, CSs are spectrally broad but close to the depletion limit without drift (i.e., for $\Omega =0$). A small decrease in the effective driving strength due to the frequency shift is thus sufficient to cross their existence limit. On the other hand, in the small detuning range, the spectral width of CSs is initially small. The effective driving strength is therefore quickly reduced by the frequency shift, all the more so as the spectral width is also narrowed by the shift.

To confirm the depletion limit from Eq. (8), we compute it by finding the corresponding saddle-node solution of Eq. (1) using a continuation scheme. The agreement is excellent except for low detunings where the homogenous background of the CS solution is not negligible.

We then look at the stability of the localised solutions. Interestingly, we find that CSs can undergo a Hopf bifurcation, denoted as ${\Omega }_{{\rm{H}}2}$, below the depletion limit. It is not found with our reduced model from the motion equations, probably because the chirp is not included in the ansatz^41^. This new bifurcation connects to the standard breathing Hopf bifurcation (${\Omega }_{{\rm{H}}1}$) that stabilises the CS solutions at low detunings. Simulations of Eq. (1) indicate that, unlike typical Hopf bifurcations, CSs do not exhibit breathing behaviour upon exceeding the frequency shift threshold ${|\Omega }_{{\rm{H}}2}|$. Instead, the oscillation keeps growing in amplitude until the soliton disappears. Hence, there is a maximum frequency shift CSs can withstand. It is set either by the depletion limit or the Hopf bifurcation. We note that for much smaller maximum slope of *V*, the $|{\Omega }_{{\rm{SL}}}|$ threshold could be reached first, as in ref. ^26^. Beyond ${\Omega }_{{\rm{SL}}}$, the existence limits given by ${\Omega }_{{\rm{DL}}}$ and ${\Omega }_{{\rm{H}}2}$ are not relevant, as they only concern steady-state CSs.

### Experimental set-up

**Fig. 2:**
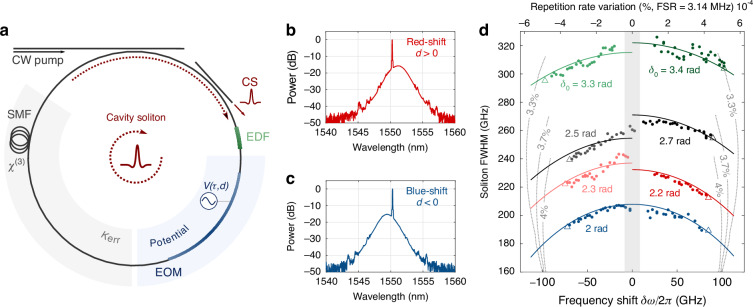
Cavity soliton central frequency manipulation. **a** Schematic of the experimental set-up. The coherently driven resonator is made of single-mode fibre (SMF) and includes an intracavity electro-optic phase modulator (EOM) to generate a sinusoidal potential $({J}_{{\rm{RF}}}=1{\rm{rad}},\,\frac{{\omega }_{{\rm{RF}}}}{2\pi }\approx 12{\rm{GHz}})$. The erbium-doped fibre (EDF) amplifier partially compensates for the cavity loss to emulate a low-loss passive resonator. Typical spectra measured for positive (**b**) or negative (**c**) potential drifts. The drift amplitude and sign control the CS spectral position, which can be blue or red-shifted with respect to the pump. **d** Spectral width of the soliton (circle), as a function of its frequency shift when the drift parameter is increased or decreased. Solitons are generated with 80 mW input power and a driving beam detuning ${\delta }_{0}$ between 2 and 3.4 rad. The last measurement before the soliton disappears is indicated by a triangle. The plain lines are the theoretical solutions from Eq. (7), and the dotted lines give the position of the Hopf ${\Omega }_{{\rm{H}}2}$ for different effective loss. Shaded area: stable CSs under an external phase modulation of the same amplitude and frequency

We aim next to confirm our theoretical predictions experimentally. A schematic of the setup is shown in Fig. 2a (see also Supplementary Fig. S4). It mainly consists of a ${L}_{{\rm{c}}}=64$ m-long fibre ring resonator (3.12 MHz free-spectral range), optically driven by a highly coherent continuous-wave (CW) laser. It incorporates an electro-optic modulator (EOM) driven by a radio-frequency harmonic signal, whose amplitude and frequency can be tuned, to generate a periodic potential. The resonator also includes a short piece of erbium-doped fibre to partially compensate for the cavity loss^42^. The measured effective loss at small signal is about 2.5%. At the driving power and detunings to observe CSs, the gain is slightly saturated by the background (the fast saturation by a single CS being negligible). Consequently, within the detuning range in the experiments, the effective loss (${\Lambda }_{{\rm{e}}}$) is estimated to lie between 3 and 4%. Beyond modulating the effective loss, we have not seen the impact of the gain.

### Temporal trapping by drifting potentials

In a first experiment, we set the driving to ${P}_{{\rm{in}}}=80$ mW and the amplitude of the IPM to ${J}_{{\rm{RF}}}=1\,{\rm{rad}}$ (i.e., $S=10.25$ and $J=54$ at 3.7% loss) to investigate the soliton properties as the drift coefficient is linearly scanned. We start with a $\frac{{\omega }_{{\rm{RF}}}}{2\pi }\approx 12{\rm{GHz}}$ frequency modulation ($W=0.47$) that matches an exact integer multiple of the cavity free spectral range (FSR) to generate a stationary phase modulation ($d=0$, see methods). Once the cavity is stabilised at a given phase detuning, we excite a single CS using a single addressing pulse^6,43^. After the transient, we slowly tune the frequency of the modulation to higher ($d < 0$) or lower ($d > 0$) values until the CS disappears. At each modulation frequency, the CS spectrum is recorded to determine the corresponding spectral width ($\propto B$) and frequency shift. Figure 2d presents the dependence of the spectral width on the frequency shift across different detuning values, encompassing both positive and negative drift regimes. A good match is observed with the analytical predictions from Eq. (7) with ${\tau }_{{\rm{s}}}={D}^{-1}{|}_{2\Omega }$, confirming the reduction of the CS spectral width with the frequency shift, that is, with the drift. The measured maximum shifts agree well with our bifurcation analysis for broad CSs (i.e., large detuning), but deviate from the value given by ${\Omega }_{{\rm{H}}2}$ for solitons at lower detunings (see also Supplementary Fig. S8). We note that Raman scattering cannot account for this discrepancy, since it should predominantly affect shorter solitons. Numerical analysis indicates that the difference likely stems from higher effective loss than expected, as the limit is highly sensitive to loss. We also compare our results to the case of external modulation. As expected from our theoretical analysis, larger shifts can be reached with internal modulations.

These first results highlight the impact of IPM on the existence and characteristics of trapped CSs. Particularly, the modulation enables to control the CS central wavelength as well as the repetition rate of the frequency comb leaving the cavity, which may open up interesting new avenues^27^. In what follows, as an illustration, we show how such control can be harnessed to overcome the detrimental effects of Raman scattering.

### Competition between temporal trapping and Raman scattering

**Fig. 3:**
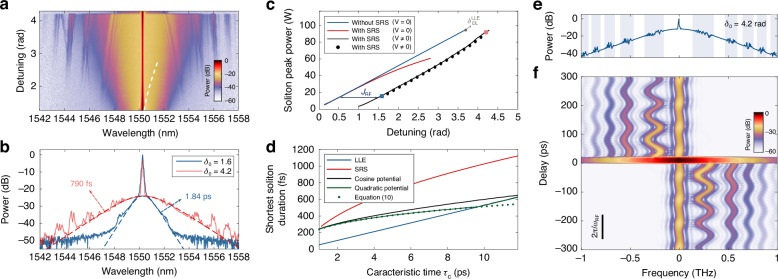
Cancellation of the Raman self-frequency shift and Bloch oscillations of Kelly sidebands. **a** Two-dimensional map of experimentally recorded spectra of a soliton trapped in a stationary intra-cavity phase modulation as a function of the detuning $({P}_{{\rm{in}}}=100{\rm{mW}},{J}_{{\rm{RF}}}=1{\rm{rad}},{\omega }_{{\rm{RF}}}/2\pi =12\,{\rm{GHz}})$. The white dashed line shows the theoretical CS central wavelength and range of existence with Raman scattering ($\bar{{\tau }_{{\rm{R}}}}=3{\rm{fs}}$) but without modulation ($V=0$). **b** Two experimental spectra corresponding to different cavity detunings ${\delta }_{0}$ (solid lines), and predicted CS spectra by numerical integration of the mean-field model. **c** Intracavity peak power of stable CSs as a function of the detuning. The black curve represents the solutions of the mean-field model with SRS (solid), and the circles indicate the result of the motion equations, both for ${\Lambda }_{{\rm{e}}}=3$%. The red and blue curves show the solutions for $V=0$ with and without SRS, respectively. We note that the black curves are shifted by the modulation amplitude ${J}_{{\rm{RF}}}$ for small detunings, i.e., small soliton bandwidths, as the CS remains located close to the minimum local detuning ${\delta }_{0}-{J}_{{\rm{RF}}}$. The squares are the experimental values reported in (**b**). **d** Shortest cavity soliton duration as a function of the characteristic time ${\tau }_{c}$ with $S=30$. Solid curves correspond to the results from numerical continuations of solutions of the mean-field model. The dots show the limit given by Eq. (10). The parameters for the potentials are ${J}_{{\rm{RF}}}/{\Lambda }_{{\rm{e}}}=105$, ${\omega }_{{\rm{RF}}}/2\pi =20{\rm{GHz}}$ and $d=0$. We note that ${\tau }_{{\rm{c}}}=7$ ps in the experiments. **e** Spectrum of a CS displaying structured Kelly sidebands (50 pm resolution) due to periodic intracavity modulation. The shadowed regions highlight the extension of the Kelly sidebands. **f** Corresponding spectrogram of the intracavity field (gate function: 20 ps-wide super-Gaussian of order 6). The vertical band at the centre is the modulated background, while the soliton spectrum is seen as the horizontal band around the zero delay

In resonators made of amorphous materials, SRS is a significant effect that may red-shifts the spectrum of cavity solitons relative to the driving laser wavelength, and whose magnitude depends on the detuning^40,44^. The frequency shift, in turn, changes the soliton repetition rate, a coupling mechanism that is beneficial to filter out the frequency noise of the driving laser, though only in a limited spectral bandwidth^45^, otherwise, it is detrimental. In addition, SRS is known to set an upper detuning limit to the CSs existence range, owing to the onset of a Hopf bifurcation. This consequently restricts their duration and bandwidth^34^. However, assuming that the SRS limitation finds its origin in the frequency shift, it should be possible to overcome the Raman limit by trapping CSs within potentials. Indeed, the synchronisation of a steady-state CS to an IPM without drift compels its spectrum to remain centred on the driving. To experimentally confirm our prediction, we use the same potential shape as in the previous experiments, but we now scan the detuning with $d=0$. Our results are shown in Fig. 3a, b. They confirm that stationary potentials do not prevent the existence of stable CSs in the presence of strong SRS. Moreover, with increasing detuning, the centre of the CS spectrum remains pinned to the driving wavelength, even as the soliton duration decreases and its spectrum widens.

In order to have a better understanding of the competition between SRS and an IPM, we first extend our previous Lagrangian analysis to include the Raman scattering by adding the term ${\tau }_{{\rm{R}}}{A}{\partial }_{\tau }{\left|A\right|}^{2}$ in the right-hand side of Eq. (1), with ${\tau }_{{\rm{R}}}$ the normalised Raman time constant (see Supplementary Information section A). We note that we here consider the linear approximation of the Raman spectral gain given the typical duration of CSs in fibre resonators (>100 fs)^46^. In the steady state, the motion equation for ${\tau }_{{\rm{s}}}$ still gives $\Omega =-d/2$. For stationary IPM, $\Omega =0$, and the equation for the frequency shift becomes:

9 $$\frac{{\rm{d}}V\left(\tau \right)}{{\rm{d}}\tau }{|}_{{\tau }_{{\rm{s}}}}={JW}\sin \left(W{\tau }_{{\rm{s}}}\right)=4{\tau }_{{\rm{R}}}{B}^{4}/15$$

It shows that the CS stabilises at a position ${\tau }_{s}$ such that the corresponding potential-induced spectral blue-shift exactly compensates the Raman soliton red-shift (see also Supplementary Figs. S6a and S7a). We note that, when progressively increasing *J* or *W*, the soliton is initially not trapped by the potential. It undergoes a Bloch oscillation until ${JW} > 4{\tau }_{{\rm{R}}}{B}^{4}/15$. Finally, the CS amplitude (*B*) is found by combining the latter equation with Eq. (7), which is unmodified by the SRS. The solutions from the motion equations are shown in Fig. 3c. An excellent agreement with the numerical continuation of the solutions of the mean-field equation is obtained. The comparison with and without IPM reveals that higher peak powers and, consequently, shorter CSs can be obtained with SRS when they are trapped in the modulation. In the experiment, we measure a 790-fs-long CS persisting endlessly in the resonator, while stable CSs are limited to 988 fs by the SRS without the potential. (We note that we have not performed measurements at $V=0$ because of the Brillouin lasing.) Yet, with the experimental parameters, the limit detuning is here set by the depletion limit (Eq. (8) for ${\Omega }_{{\rm{DL}}}=0$). The shortest CS is thus identical to the one given by the LLE.

Equation (9) suggests that the LLE limit could always be reached, provided that the potential is sufficiently steep. To broaden our analysis, we investigate how far the balance between the Raman red shift and the potential-induced blue shift can be maintained. We find that at large detunings, CSs can undergo a new saddle-node bifurcation before reaching the depletion limit. Figure 3d shows for $S=30$ the duration of the shortest stable CS as a function of the cavity characteristic time, ${\tau }_{{\rm{c}}}=\sqrt{-{\beta }_{2}{L}_{{\rm{c}}}/{\Lambda }_{{\rm{e}}}}$, for a large amplitude harmonic IPM, as well as for its parabolic approximation $V\left(\tau \right)=-J[1-{\left(W\tau \right)}^{2}/2]$. These results are compared to the corresponding shortest CS without trapping, both in the absence and presence of SRS. We see that the modulation enables the CS to approach or even reach the standard limit without SRS. Yet, as ${\tau }_{{\rm{c}}}$ decreases, the compensation of the self-frequency shift becomes less effective. Ultimately, the confinement in the IPM does not suffice to stabilise the CS beyond the Raman limit (not shown). Interestingly, for stationary quadratic modulations, the motion equations can be solved to find the shortest soliton width (See Supplementary Information section C). In dimensional units, it reads:

10 $$\Delta {\tau }_{{\rm{FWHM}},\min }\approx 2.02{\left(\frac{{\Lambda }_{{\rm{e}}}{\bar{{\tau }_{{\rm{R}}}}}^{2}{\tau }_{{\rm{c}}}^{2}}{2{J}_{{\rm{RF}}}{\omega }_{{\rm{RF}}}^{2}}\right)}^{\frac{1}{6}}$$

where $\bar{{\tau }_{{\rm{R}}}}={\tau }_{{\rm{R}}}{\tau }_{{\rm{c}}}$ is the Raman time constant. An excellent agreement with the numerical solutions of the mean-field model is obtained, providing that the Raman compensation is the limiting factor.

### Bloch oscillations of the Kelly sidebands

The spectrum reported in Fig. 3 at high detuning deviates significantly from that of a sech-shape soliton, exhibiting broad and highly structured features in its wings. Numerical simulations of trapped CSs with a lumped-element model of our cavity, show that they originate from periodic perturbations over the cavity round-trip (see Fig. 3e). Without the internal phase modulation, these perturbations give rise to narrowband spectral peaks, known as Kelly sidebands. Their location satisfy the quasi phase-matching relation $\left[\beta \left(\omega \right)-\beta \left({\omega }_{0}\right)-{\beta }_{1}\times \left(\omega -{\omega }_{0}\right)\right]{L}_{{\rm{c}}}=2\pi m+{\delta }_{0}$, where *β* is the propagation constant, ${\beta }_{1}$ is the inverse of the group velocity, ${\omega }_{0}$ is the CS central frequency, ${\delta }_{0}$ is the cavity detuning and *m* is an integer. These peaks are here broadened by the electro-optic modulator. Yet, the modulation does not produce regular sidebands, nor their width and location can be predicted by a generalised phase-matching condition in which ${\delta }_{0}\to {\delta }_{0}+{\phi }_{\mathrm{int}}(\tau )$ with ${\phi }_{\mathrm{int}}(\tau )$ the IPM (see Supplementary Information section A). To gain more insight, Fig. 3f shows the spectrogram of the intracavity field. It reveals that the complex shape of the Kelly-bands in the spectrum comes from the interference of very regular oscillations of the Kelly waves in the time-frequency domain. These oscillations originate in the relative drift between the linear waves and the IPM. They can be seen as Bloch oscillations (BOs) along a synthetic frequency dimension^47^. However, contrary to BO experiments with linear wave packets in recirculation loops^47^ or CSs in driven resonators^26^, the linear (Kelly) waves are here continuously generated by the soliton, resulting in a stationary pattern, round-trip after round-trip. The effective force $F$ responsible for the oscillatory motion in the synthetic frequency lattice is $F$ $=\Delta \nu /(n\times {\rm{FSR}})$, where $\Delta \nu =n\times {\rm{FSR}}-{{\rm{\nu }}}_{{\rm{RF}}}$ with ${{\rm{\nu }}}_{{\rm{RF}}}$ the frequency of the phase modulation and *n* the closest integer multiple between ${\nu }_{{\rm{RF}}}$ and the FSR^26^. Yet, owing to the dispersion, for a stationary phase modulation at ${\omega }_{0}$ ($d=0$), $F$ is directly proportional to $\Delta \omega =\omega -{\omega }_{0}$, where *ω* is the frequency of the sideband. Since the amplitude of BOs is given by ${A}_{{\rm{BO}}}={J}_{{\rm{RF}}}/$ $F$, this accounts for the reduction in the amplitude of the spectral oscillations with the distance of the spectral sideband from the central CS frequency seen in Fig. 3f. More precisely, the oscillation amplitudes from the simulation agree very well with the $1/\Delta \omega$ scaling expected for BOs (see Supplementary Information section D). These dynamics also explain the asymmetry between modulated sidebands of the same order. The interplay with Raman scattering shifts the stationary position of the CS away from the potential minimum, leading to different phase shifts in the oscillations of the leading and trailing emitted waves, which in turn give rise to an asymmetric spectral interference pattern. We note that in the time domain, the Kelly sidebands produce oscillatory tails on the soliton that mediate long-range bound states between CSs^48^. The Bloch oscillations of the Kelly waves alter the tail pattern; however, it remains stationary, meaning that CSs can still interact through their tails. The corresponding long-range oscillating interaction potential adds to the periodic potential and could modify the position of solitons individually trapped at different locations (see Supplementary Fig. S7).

## Discussion

In this work, we have studied how the unique particle-like characteristics of solitons and their interaction with slowly varying trapping potentials can be leveraged to control the properties of cavity solitons. In our experiments, the potential is generated by IPM at a frequency close to a high harmonic of the FSR of our fibre resonator. We have shown that the locking of the cavity soliton to the modulation enables to up- or down-shift its spectrum with respect to the driving frequency by desynchronising the modulation to the cavity round-trip time. The CS then settles at a position in the modulation reference frame offset from the minimum, where a linear temporal phase is applied across the pulse at each pass. This enables the photons to be slightly up- or down-shifted by a Doppler-like frequency shift^49^ (also called spectral shearing^50,51^). The interplay between the driving, four-wave mixing and the single-pass frequency shift introduced by the modulator results in a finite spectral shift of the CS relative to the driving.

We theoretically found that for deep potentials, the limit spectral shift ($\delta {\omega }_{\max }$) is fundamentally set either by the driving pump depletion limit or a Hopf bifurcation, while it is set by the maximum potential slope for shallow potentials^26^. The maximum spectral shift, in turn, sets a limitation on the tuning range of the output soliton comb repetition rate: ${\rm{\Delta }}{t}_{{\rm{R}}}^{\max }/{t}_{{\rm{R}}}=2\left|{\beta }_{2}\right|{L}_{{\rm{c}}}{\rm{FSR}}\,\delta {\omega }_{\max }$, where the factor of 2 arises because the soliton can experience either a blue shift or a red shift.

Experimentally, we have investigated the trapping by intracavity periodic phase modulations. We have observed that the soliton spectrum can robustly be spectrally blue- or red-shifted and that the reduction of the CS amplitude with the frequency shift is very well predicted by our theoretical model. The measured limit shifts broadly align with the bifurcation analysis but are too small for narrowband solitons, likely due to background-induced gain saturation in our setup.

We then demonstrated that the ability to deterministically manipulate the soliton frequency can be exploited to overcome the soliton self-frequency shift arising from the SRS. We observed the formation of potential-stabilised solitons that would not otherwise have existed^34^. However, we theoretically showed that there is a minimum soliton duration below which the balance between the potential induced blue-shift and the Raman red-shift cannot be maintained.

Previous works have shown that cavity solitons can also be trapped and manipulated by external phase modulations^17,19^ of the coherent driving. Yet, compared to external modulations, IPMs enhance the tuning range of the CS spectral shift and thus of the soliton comb repetition rate. In our experiment, the maximum shifts correspond to a relative variation range of the comb repetition rate of $5.5\times {10}^{-6}$. This represents a 50-fold improvement over previous demonstrations in a fibre resonator using external phase modulation^17^ and a tenfold improvement with our cavity parameters. The internal modulation also increases the CS speed in response to a phase gradient, in proportion to the inverse of the cavity loss ${\Lambda }_{{\rm{e}}}$. The improvement can be significant for low-loss resonators, highlighting the importance of dissipation in the dynamics of CSs in internally modulated resonators. This is of prime interest for applications to micro-comb given the recent developments of on-chip high-finesse lithium niobate resonators that incorporate a high-speed electro-optic modulator^27^ eliminating the need for an amplifier, as required in this work.

We envision intracavity phase trap to play an important role in the future of cavity soliton based frequency combs. On the one hand, the robust locking mechanism can be leveraged for generating ultra low-noise optical combs and microwave signals through direct synchronisation of the cavity solitons to an external microwave reference^27^ or by employing a coupled optoelectronic oscillator scheme^52–54^. On the other hand, the motion equations show that the potential gradient governs the rate at which the soliton frequency can be changed per round trip. The ability to rapidly vary the soliton frequency and, thus, the soliton comb repetition rate by steep potentials can benefit many applications, such as LiDAR and sensing. Finally, our findings could shed light on recently reported results in internally modulated cavity soliton microcombs^27^, and could be extended to solitary waves of the parametrically driven nonlinear Schrödinger equation in optics^55,56^ and hydrodynamics^3^, where the potential will interact with the attraction or repulsion force in soliton pairs^57^.

## Materials and methods

### Numerical continuation

We performed numerical continuations using the BifurcationKit Julia library^58^. We discretized the mean-field equations onto a $N=512$ points lattice on a time window that entirely contains the soliton. Numerical derivatives were computed in the spectral domain (multiplication by ${\left(-i\omega \right)}^{n}$ for *n*th order derivative). From this, we implemented a fully matrix-free Jacobian-Vector product (JvP). Similarly, we used a matrix-free generalised minimal residual iterative method for solving the JvP linear systems that arise for every Newton-Raphson steps. We preconditioned the linear solver by the inverse of the derivatives spectral representation ($1/{\left(-i\omega \right)}^{n}$), as we found that it greatly accelerated the convergence rate. Continuation of bifurcation points (Hopf and SN) was achieved with the Minimally Augmented formulation^59^. Numerical convergence with a higher number of points *N* was verified.

### Experimental set-up

The experimental set-up used to investigate the dynamics of cavity solitons in trapping potentials consists of an active fibre ring resonator^42^ of length ${L}_{{\rm{c}}}=64\,{\rm{m}}$ (free spectral range of 3.12 MHz, ${\tau }_{c}=7\,{\rm{ps}}$). Specifically, it is made of 63 m of standard telecommunication single-mode fibre (SMF-28, $\gamma =1.3\,{{\rm{W}}}^{-1}{\rm{k}}{{\rm{m}}}^{-1},{\beta }_{2}=-2.3\times {10}^{-26}{{\rm{s}}}^{2}{{\rm{m}}}^{-1}$) spliced to a 75 cm-long segment of erbium-doped fibre (EDF, Liekki® ER16-8/125). The aim of the doped fibre is to partially compensate for the high cavity roundtrip loss ($T=-2.8$ dB when the EDF is removed, corresponding to an intrinsic finesse of ${\mathscr{F}}=9.7$). The length of the amplifying section has been carefully adjusted following the method described in ref. ^42^. The EDF is pumped by a 1480 nm laser through a wavelength division multiplexer, with a power ${P}_{{\rm{p}}}^{{\rm{in}}}=2$ W. The unabsorbed pump power ${P}_{{\rm{p}}}^{{\rm{out}}}$ after the EDF is rejected by a second WDM. In addition, to individually address a soliton by means of a single writing pulse^6,43^, the cavity includes a 1535/1550 WDM. The writing pulses come from a 1535 nm mode-locked laser and are gated using an acousto-optic modulator (see Supplementary Fig. S3). The three WDMs combined spectral transmission prevents lasing at shorter wavelengths^43^. With intracavity amplification, the measured effective loss at small signal is about ${\Lambda }_{{\rm{e}}}=2.5 \%$ (effective finesse ${{\mathscr{F}}}_{{\rm{e}}}=250$). The saturation power is estimated to 250 mW from the numerical model developed in ref. ^42^. In the mean-field simulations and numerical continuations, the gain saturation was not included explicitly but accounted for by using a higher effective loss. The resonator includes an electro-optic phase modulator to generate the (real) potential. The EOM is driven by a harmonic RF signal whose amplitude (${J}_{{\rm{RF}}}$) and frequency (${\nu }_{{\rm{RF}}}$) can be freely chosen. ${J}_{{RF}}$ is experimentally limited to 1 rad at the modulation frequency ($\approx 12\,{\rm{GHz}}$). The optical cavity is coherently driven with a sub-100-Hz continuous wave laser, centred on 1550.12 nm. It is amplified by a commercial erbium-doped fibre amplifier (EDFA) before being injected into the cavity through the 90/10 input coupler. The driving beam polarisation is adjusted at the cavity input with a polarisation controller to excite one eigen polarisation mode of the cavity. Part of the intracavity power is extracted at the output coupler (99/1) to perform spectral measurements of the intracavity field. We note that the IPM prevents the build-up of stimulated Brillouin scattering. Therefore, there is no need for an optical isolator, even if the cavity is pumped by a highly coherent CW laser. This enables sending a co-polarised frequency-shifted counter-propagating CW control signal to stabilise the cavity detuning. Its polarisation is identical to the driving beam to avoid large insertion loss in the EOM. The cavity detuning is actively stabilised by using a proportional-integral-derivative (PID) controller fed by the signal generated by the output control beam on a photodiode (200 kHz bandwidth). The cavity detuning is tuned by slightly shifting the frequency of the control signal with a frequency shifter (FS).

### Drifting potential

To study how CSs behave in a drifting potential (Fig. 2b–e), we first precisely stabilise the cavity detuning Δ by means of the frequency shifter (FS). Once the CS is excited by a single writing pulse, we change the frequency modulation by steps of 1 kHz and simultaneously record the optical spectrum with an optical spectrum analyser (OSA) until the soliton disappears. We repeat the experiment three times for each detuning to ensure that the soliton disappearance is related to the potential drift and not the consequence of an external perturbation.

### Raman cancellation

The experimental procedure is similar to the one for drifting potentials, except that the modulation is maintained equal to an integer multiple of the free spectral range ($d=0$).

## Supplementary information

Supplementary information file

## Data Availability

The data that support the findings of this study are available from the corresponding author upon reasonable request.

## Code availability

The code used for simulation and plotting of results is available upon request from the corresponding author.
