# Supplementary material for: Dynamics of driven dissipative temporal solitons in an intracavity phase trap
Source: Light Sci Appl. 2026 Feb 18;15:117. doi: 10.1038/s41377-025-02147-8 (PMC12913624; doi:10.1038/s41377-025-02147-8)
Supplement: Supplementary file 1 — Supplementary information file [file 41377_2025_2147_MOESM1_ESM.pdf]

Supplementary information – Dynamics of driven dissipative temporal solitons in an intracavity phase trap

Nicolas Englebert,[1,2] Corentin Simon,[1] Carlos Mas Arabí,[1,3] François Leo,[1] and Simon-Pierre Gorza[1,*]

[1]*Service OPERA-Photonics, Université libre de Bruxelles (U.L.B.), 50 Avenue F. D. Roosevelt, CP 194/5, B-1050 Brussels, Belgium*

[2]*Department of Electrical Engineering, California Institute of Technology, Pasadena, California 91125, USA*

[3]*Institut Universitari de Matemàtica Pura i Aplicada, Universitat Politècnica de València, 46022 (València), Spain*

Contents

A. Modelling - Driven-dissipative Gross-Pitaevskii equation 1
1. Dimensional form 1
2. Normalized form 2
B. Reduced model – Equations of motion 2
1. Dimensional form 2
2. Normalized form 3
C. Cancellation of the Raman-induced self-frequency shift by external potentials: limit of shortest CSs 3
D. Bloch oscillations of the Kelly bands 4
E. Internal versus external phase modulation 5
F. Supplementary Figures 6

References 9

A. Modelling - Driven-dissipative Gross-Pitaevskii equation

1. Dimensional form

The dynamics of Kerr cavity solitons (CSs) in trapping potentials $\overline{V}$ can be described by a Lugiato-Lefever equation (LLE) [1, 2], generalized to account for the intracavity phase modulation, $\phi_{\text{int}} = -\overline{V}$, [3] and the stimulated Raman scattering (SRS) [4, 5]. Such generalization corresponds to a driven-dissipative Gross-Pitaevskii equation, which reads:

$$it_{\text{R}}\frac{\partial E}{\partial t} = \left(\left[\delta_0 + \overline{V}(\tau)\right] - i\frac{\Lambda_{\text{e}}}{2} + i\left[t_{\text{R}}\bar{d}\frac{\partial}{\partial\tau} - i\frac{\beta_2 L_{\text{c}}}{2}\frac{\partial^2}{\partial\tau^2}\right] - \gamma L_{\text{c}}\left[|E|^2 - \overline{\tau_{\text{R}}}\frac{\partial|E|^2}{\partial\tau}\right]\right)E + i\sqrt{\theta P_{\text{in}}} \tag{S1}$$

where $t_{\text{R}} = \text{FSR}^{-1}$ is defined as the inverse of the cavity free spectral range (FSR) and corresponds to the cavity roundtrip time at the driving wavelength. t is the (slow) time describing the evolution of the electric field envelope $E(t,\tau)$ with τ, a (fast) time variable defined in a co-moving reference frame in which the potential $\overline{V}(\tau)$ is stationary with time t. $\Lambda_{\text{e}} = -\ln(T \times G)$ denotes the effective cavity loss that takes into account the "cold cavity" transmission (T) and the intracavity gain of the optical amplifier (amplification factor G) [6]. The phase detuning between the driving field and the closest cavity resonance is $\delta_0 = 2\pi m - \beta L_{\text{c}}$, with L_{c}, the cavity length, β, the propagation constant, and m, an integer. β_2 and γ are, respectively, the group-velocity dispersion and the nonlinear Kerr coefficient of the resonator. $\overline{\tau_{\text{R}}}$ is the Raman time constant. θ and P_{in} are the input coupler ratio and coherent driving power, respectively. Finally, $\bar{d}$ is the drift coefficient of a wave at the driving frequency in the reference frame co-moving with the potential. For an intracavity phase modulation of the form $J_{\text{RF}}\cos(\omega_{\text{RF}}t)$, we thus have $\overline{V} = -J_{\text{RF}}\cos(\omega_n\tau)$ and $\bar{d} = \Delta\omega/\omega_n$, where $\Delta\omega = \omega_n - \omega_{\text{RF}}$ is the frequency difference between the nth harmonic of the cavity FSR ($\omega_n = n \times 2\pi \times \text{FSR}$) and the modulation frequency.

*Electronic address: simon.pierre.gorza@ulb.be

2. Normalized form

It is convenient to introduce dimensionless parameters to carry out theoretical analyses and draw general conclusions. Choosing the usual LLE normalization with $\tau_\mathrm{c} = \sqrt{-\beta_2 L_\mathrm{c}/\Lambda_\mathrm{e}}$ and $\alpha_\mathrm{e} = \Lambda_\mathrm{e}/2$ [2]:

$$t \to \frac{\alpha_\mathrm{e} t}{t_\mathrm{R}}, \qquad \tau \to \frac{\tau}{\tau_\mathrm{c}} \qquad A = E\sqrt{\frac{\gamma L_\mathrm{c}}{\alpha_\mathrm{e}}} \qquad S = \sqrt{\frac{\gamma L_\mathrm{c}\theta_\mathrm{in} P_\mathrm{in}}{\alpha_\mathrm{e}^3}}$$
$$\Delta = \frac{\delta_0}{\alpha_\mathrm{e}}, \qquad V = \frac{\overline{V}}{\alpha_\mathrm{e}} \qquad d = \bar{d}\frac{t_\mathrm{R}}{\tau_\mathrm{c}\alpha_\mathrm{e}} \qquad \tau_\mathrm{R} = \frac{\overline{\tau_\mathrm{R}}}{\tau_\mathrm{c}} \tag{S2}$$

Eq. (S1) becomes:

$$i\frac{\partial A(t,\tau)}{\partial t} = iS + \left(-i + \Big[\Delta + V(\tau)\Big] - \left[|A|^2 - \tau_\mathrm{R}\frac{\partial |A|^2}{\partial \tau^2}\right] + i\left[d\frac{\partial}{\partial \tau} + i\frac{\partial^2}{\partial \tau^2}\right]\right) A \tag{S3}$$

which corresponds to Eq. (1) of the main manuscript when $\tau_\mathrm{R} = 0$.

B. Reduced model – Equations of motion

1. Dimensional form

There are no known closed-form analytical expressions that would describe CS solutions of the mean-field equation (S1), with or without the SRS term. However, equations of motion can be found by using a perturbative Lagrangian approach [7]. Starting with the soliton ansatz [5, 7]:

$$E_s(t,\tau) = \overline{B}\,\mathrm{sech}\left(\frac{\overline{B}(\tau - \tau_\mathrm{s})}{\sqrt{-\beta_2/\gamma}}\right) \mathrm{e}^{-i\overline{\Omega}(\tau-\tau_\mathrm{s})}\mathrm{e}^{i\overline{\phi}} \tag{S4}$$

where $\overline{\Omega}$ corresponds to the soliton central frequency, $\overline{B}$ is the soliton amplitude (with units of $\mathrm{W}^{1/2}$), τ_s is the soliton position along the fast-time, and $\overline{\phi}$ is the cavity soliton phase. Following the procedure described in [5, 7, 8] with the linear approximation of the potential around τ_s, one can find the equations of motion for each of the CS (dimensional) parameters:

$$\begin{aligned}
t_\mathrm{R}\frac{\mathrm{d}\overline{\Omega}}{\mathrm{d}t} &= -\frac{\overline{\Omega}}{\overline{B}} t_\mathrm{R}\frac{\mathrm{d}\overline{B}}{\mathrm{d}t} - 2\alpha_\mathrm{e}\overline{\Omega} + \frac{\mathrm{d}\overline{V}(\tau_\mathrm{s})}{\mathrm{d}\tau_\mathrm{s}} + \frac{8\overline{\tau_\mathrm{R}}\gamma^2 L_\mathrm{c}}{15\beta_2}\overline{B}^4 \\
t_\mathrm{R}\frac{\mathrm{d}\overline{B}}{\mathrm{d}t} &= -2\alpha_\mathrm{e}\overline{B} + \pi\sqrt{\theta P_\mathrm{in}}\cos(\overline{\phi})\,\mathrm{sech}\left(\sqrt{\frac{-\beta_2}{\gamma}}\frac{\overline{\Omega}\pi}{2\overline{B}}\right) \\
t_\mathrm{R}\frac{\mathrm{d}\tau_\mathrm{s}}{\mathrm{d}t} &= \beta_2 L_\mathrm{c}\overline{\Omega} - t_\mathrm{R}\bar{d} + \frac{\pi^2}{2\overline{B}^2}\sqrt{\frac{-\beta_2}{\gamma}\theta P_\mathrm{in}}\sin(\overline{\phi})\,\mathrm{sech}\left(\sqrt{\frac{-\beta_2}{\gamma}}\frac{\overline{\Omega}\pi}{2\overline{B}}\right)\tanh\left(\sqrt{\frac{-\beta_2}{\gamma}}\frac{\overline{\Omega}\pi}{2\overline{B}}\right) \\
t_\mathrm{R}\frac{\mathrm{d}\overline{\phi}}{\mathrm{d}t} &= \frac{\gamma L_\mathrm{c}\overline{B}^2}{2} + \frac{\beta_2 L_\mathrm{c}}{2}\overline{\Omega}^2 - [\delta_0 + \overline{V}(\tau_\mathrm{s})] - t_\mathrm{R}\overline{\Omega}\left[\frac{\mathrm{d}\tau_\mathrm{s}}{\mathrm{d}t} + \bar{d}\right] \\
&\quad - \frac{\pi^2}{2\overline{B}^2}\overline{\Omega}\sqrt{\frac{-\beta_2}{\gamma}\theta P_\mathrm{in}}\sin(\overline{\phi})\,\mathrm{sech}\left(\sqrt{\frac{-\beta_2}{\gamma}}\frac{\overline{\Omega}\pi}{2\overline{B}}\right)\tanh\left(\sqrt{\frac{-\beta_2}{\gamma}}\frac{\overline{\Omega}\pi}{2\overline{B}}\right)
\end{aligned} \tag{S5}$$

2. Normalized form

Applying the normalization Eq. (S2) to the system of equations (S5) and neglecting the terms proportional to $\overline{B}^{-2}$ yield Eqs. (2)-(5) in the main manuscript (for $\tau_{\mathrm{R}} = 0$):

$$\begin{aligned}
\frac{\mathrm{d}\Omega}{\mathrm{d}t} &= -\frac{\Omega}{B}\frac{\mathrm{d}B}{\mathrm{d}t} - 2\Omega + \frac{\mathrm{d}V(\tau_{\mathrm{s}})}{\mathrm{d}\tau_{\mathrm{s}}} - \frac{4\tau_{\mathrm{R}}}{15}B^4 \\
\frac{\mathrm{d}B}{\mathrm{d}t} &= -2B + \pi S\cos(\phi)\,\mathrm{sech}\left(\frac{\Omega\pi}{\sqrt{2}B}\right) \\
\frac{\mathrm{d}\phi}{\mathrm{d}t} &= \frac{B^2}{2} - \Omega^2 - [\Delta + V(\tau_{\mathrm{s}})] - \left(\frac{\mathrm{d}\tau_{\mathrm{s}}}{\mathrm{d}t} + d\right)\Omega \\
\frac{d\tau_{\mathrm{s}}}{dt} &= -2\Omega - d
\end{aligned} \tag{S6}$$

C. Cancellation of the Raman-induced self-frequency shift by external potentials: limit of shortest CSs

From the motion equations, we can find the conditions under which a potential completely cancels the Raman-induced frequency shift of cavity solitons. To do so, we look at the stationary solutions at $\Omega = 0$ (we note that red- and blue-shifted CSs still exist in the presence of SRS, but we here restrict ourselves to unshifted solutions). The stationary solution of S6 reads:

$$\begin{aligned}
&\Omega = d = 0 \\
&\cos(\phi) = \frac{2B}{\pi S} \\
&\frac{B^2}{2} = \Delta + V(\tau_{\mathrm{s}}) \\
&\mathcal{D}(\tau_{\mathrm{s}}) = \frac{4\tau_{\mathrm{R}}}{15}B^4
\end{aligned} \tag{S7}$$

where τ_{s} is the position of the CS in the fast time and $\mathcal{D}(\tau_{\mathrm{s}}) = \frac{dV(\tau)}{d\tau}|_{\tau_{\mathrm{s}}}$ is the local potential gradient.

First, the motion equation for the CS amplitude gives $\cos(\phi) = \frac{2B}{\pi S} < 1$, which corresponds to the depletion limit. It implicitly accounts for the local detuning $\Delta_{\mathrm{s}} = \Delta + V(\tau_{\mathrm{s}})$ through the soliton amplitude B.

A second existence condition arises from the combination of the two last equations:

$$\Delta = \frac{B^2}{2} - V\left[\mathcal{D}^{-1}\left(\frac{4\tau_{\mathrm{R}}}{15}B^4\right)\right] \tag{S8}$$

where $\mathcal{D}^{-1}$ is the reciprocal of the potential gradient. We now focus on trapping by parabolic potentials of the form $V(\tau) = a\tau^2/2 > 0$, for which there is no slope limit and an explicit analytical solution can be found. We note that we do not consider any offset on the potential since this term simply shifts the detuning $[\Delta \to \Delta + V(0)]$. We thus have $V\left[\mathcal{D}^{-1}(x)\right] = \frac{x^2}{2a}$, which yields:

$$\Delta = \frac{B^2}{2} - \frac{1}{2a}\left(\frac{4\tau_{\mathrm{R}}}{15}\right)^2 B^8 = \frac{B^2}{2} - \frac{1}{8}\frac{B^8}{B_{\mathrm{max}}^6} \tag{S9}$$

where we have introduced $B_{\mathrm{max}} = (225a/64\tau_{\mathrm{R}}^2)^{1/6}$ for convenience (see below). The right-hand side of this later equation shows that there is a maximum possible value for Δ (see also Fig.1). It is found by looking at the soliton amplitude such that $\partial\Delta/\partial B = 0$, which occurs at $B = B_{\mathrm{max}}$ and $\Delta = \Delta_{\mathrm{max}} = (3/8)B_{\mathrm{max}}^2$. This gives the maximum peak power B_{max}^2 of stable CSs for which the interaction with the intracavity phase modulation can cancel the SRS. We note that this limit is independent of the driving amplitude S. In conclusion, there are two limitations:

$$B < B_{\mathrm{max}} \quad \text{and} \quad B < \frac{\pi S}{2} \tag{S10}$$

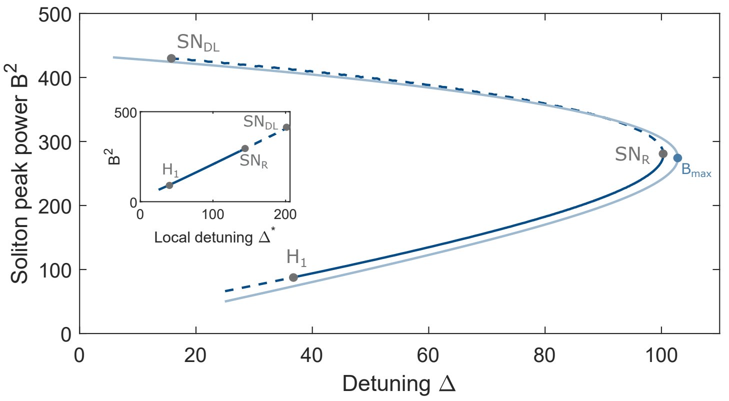

Fig. S1. Peak power of the cavity solitons as a function of the detuning Δ with a trapping parabolic potential and when considering the Raman self-frequency shift. The parameters are $\tau_\mathrm{R} = 1.5 \times 10^{-3}$ ($\overline{\tau_\mathrm{R}} = 3\,\mathrm{fs}$[4], $\tau_\mathrm{c} = 2\,\mathrm{ps}$), $S = 30$, and $a = 13.2$ [parabolic approximation of a cosine modulation at frequency $\mathcal{W} = 0.25$ ($\nu_\mathrm{RF} = 20\,\mathrm{GHz}$) and $J = 209$]. The dark blue line shows results from the numerical continuations of solutions of the mean-filed model, where unstable solutions are indicated in dashed lines. H_1, Hopf bifurcation that stabilizes the solution. The two saddle-node bifurcations, SN_R and SN_DL, correspond to the conditions $B = B_\mathrm{max}$ and $B = \pi S/2$, respectively. For detunings Δ below SN_R, there are two different soliton solutions, with different positions τ_s and local detuning Δ_s (see also inset), but only one is stable. Equation (S9) is plotted in light blue. We note a very good agreement between the two models regarding the peak power (and thus the soliton duration) at the saddle-node SN_R.

Looking at the SRS limitation and applying the denormalization $a \rightarrow \overline{a}\tau_\mathrm{c}^2/\alpha_\mathrm{e}$ [see also Eq. (S2)], the corresponding minimum CS duration in dimensional units reads:

$$\mathrm{FWHM}_\mathrm{min} = 4\sqrt{2}\ln\left(1+\sqrt{2}\right)\sqrt[3]{\frac{1}{15}}\left(\frac{\alpha_\mathrm{e}\tau_\mathrm{c}^2\overline{\tau_\mathrm{R}}^2}{\overline{a}}\right)^{1/6} \approx 2.02\left(\frac{\alpha_\mathrm{e}\tau_\mathrm{c}^2\overline{\tau_\mathrm{R}}^2}{\overline{a}}\right)^{1/6} \tag{S11}$$

Considering the parabolic approximation of a cosine potential of the form $\overline{V} = -J_\mathrm{RF}\cos(\omega_\mathrm{RF}\tau)$, we have $\overline{a} = J_\mathrm{RF}\omega_\mathrm{RF}^2$. It follows Eq.(9) of the main manuscript. This limit is in excellent agreement with the results of the numerical continuations (Figure S1) of the solutions of the mean-field model. We finally note that exact cosine potentials can also be solved. In this case, finding the maximum CS amplitude for which $\partial\Delta/\partial B = 0$ requires computing the unique positive root of a third-order polynomial.

D. Bloch oscillations of the Kelly bands

The electro-optic phase modulator included in the fiber loop introduces a coupling between the modes of the resonator with a strength given by $J = J_\mathrm{RF}/(2t_\mathrm{R})$ for a modulation $J_\mathrm{RF}\cos(2\pi\nu_\mathrm{RF}\tau)$, where ν_RF is a frequency close to an integer multiple (n) of the FSR. These coupled sites form a synthetic 1D lattice along the frequency axis [8]. By detuning the modulation from a resonant frequency ($\nu_\mathrm{RF} = n\times\mathrm{FSR} + \Delta f$), an effective force along the frequency axis is implemented. This force, which is responsible for the Bloch oscillations (BOs) in the synthetic frequency lattice, is given by:

$$F = \dot{d} = \frac{n\times\mathrm{FSR} - \nu_\mathrm{RF}}{n\times\mathrm{FSR}} \tag{S12}$$

Assuming that the modulation is resonant at the driving frequency ω_0 ($\nu_\mathrm{RF} = n_0 \times \mathrm{FSR}|_{\omega_0}$), this modulation becomes detuned from resonance at ω because of chromatic dispersion. We thus have $\Delta f = n_0(\mathrm{FSR}|_{\omega_0} - \mathrm{FSR}|_{\omega}) \approx \frac{\beta_2}{\beta_1}n_0\mathrm{FSR}|_{\omega_0}\times(\omega-\omega_0)$. The effective force thus reads:

$$F \approx \frac{\Delta f}{\nu_\mathrm{RF}} = \frac{\beta_2}{\beta_1}\Delta\omega \tag{S13}$$

where $\Delta\omega = \omega - \omega_0$.

BOs are characterized by an oscillation amplitude $A_{\rm BO} = 2J/F$. This amplitude thus reads:

$$A_{\rm BO}|_\omega = \frac{J_{\rm RF}\beta_1}{t_{\rm R}\beta_2}\frac{1}{\Delta\omega} = \frac{J_{\rm RF}}{L_{\rm c}\beta_2}\frac{1}{\Delta\omega} \tag{S14}$$

with $t_{\rm R} = \beta_1 L_{\rm c}$.

The amplitude of the BOs as a function of the inverse of the frequency shift from the driving is shown in figure 2. The circles denote the data measured from the numerical simulation of the dynamics of CS with a periodic potential, which is reported in Fig.7 of the main manuscript. These results are in excellent agreement with the theoretical model Eq. (S14), confirming that the oscillations of the Kelly bands can be interpreted as Bloch oscillations.

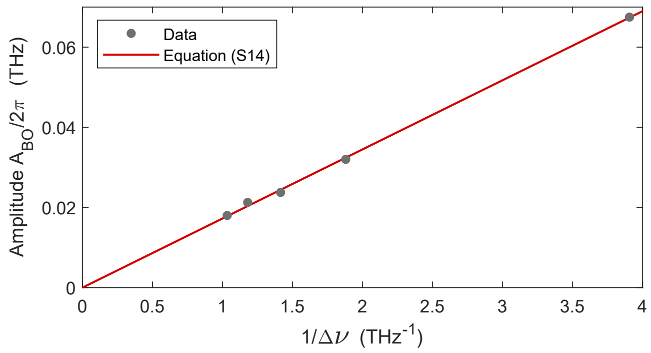

Fig. S2. Amplitude of the spectral oscillations of the Kelly sidebands as a function of the inverse of their frequency shift from the driving ($\Delta\nu = \Delta\omega/2\pi$). The circles are the amplitudes extracted from the numerical simulation shown in Fig.7 of the main manuscript. The parameters are $\beta_2 = -2.3\times10^{-26}\,{\rm s^2m^{-1}}$, $L_{\rm c} = 64\,{\rm m}$. The red line shows the theoretical relation Eq.S14.

E. Internal versus external phase modulation

Studies of the motion dynamics of cavity solitons in presence of a modulated driving field, either in phase or in amplitude, have revealed that cavity solitons can be manipulated and trapped by the modulations [9–12]. For instance, such trapping as been used to control and stabilize the soliton repetition rate [13], which is interesting for applications. Real potentials, as considered in this work, correspond to phase modulations of the intracavity field. Of particular interest for comparison are, therefore, external purely phase-modulated driving fields.

For an *external* phase modulation, the CS drift velocity in normalized variables reads [12]:

$$v_{\rm ext} = \frac{d\tau_{\rm s}}{dt} = 2\phi'_{\rm ext} - d \tag{S15}$$

where ' stands for the first derivative with respect to the fast-time τ. The corresponding dimensional expression of the drift velocity is:

$$\overline{v}_{\rm ext} = \frac{L_{\rm c}}{t_{\rm R}}|\beta_2|\frac{d\phi_{\rm ext}}{d\tau}|_{\tau_{\rm s}} - \overline{d}. \tag{S16}$$

This can be compared with Eq. (5) in the main manuscript for *internal* phase modulations:

$$v_{\rm int} = -2\Omega - d \tag{S17}$$

with Ω the normalized frequency shift of the CS. Hence, this gives for $\overline{V} = -\phi_{\rm int}$:

$$\overline{v}_{\rm int} = \frac{1}{\Lambda_{\rm e}}\frac{L_{\rm c}}{t_{\rm R}}|\beta_2|\frac{d\phi_{\rm int}}{d\tau}|_{\tau_{\rm s}} - \overline{d} \tag{S18}$$

We can see that the equations (S16) and (S18) are identical, providing that

$$\phi_{\rm int} = \Lambda_{\rm e}\phi_{\rm ext} \tag{S19}$$

This result shows that a phase modulation amplitude $1/\Lambda_e$ times smaller is required for internally modulated resonators to achieve the same effect as with external modulation of the driving beam. The locking range, and hence the maximum frequency shift of the CS or the tuning range of the soliton repetition rate, is thus enhanced by the same factor, which can also be written $\mathcal{F}/2\pi$ or $Q\nu_0/(2\pi \times \mathrm{FSR})$, with Q the resonator Q-factor and ν_0 the soliton carrier frequency.

This emphasizes the importance of the loss in the dynamics of CSs in internally modulated resonators as well as the interest of internal modulations for applications.

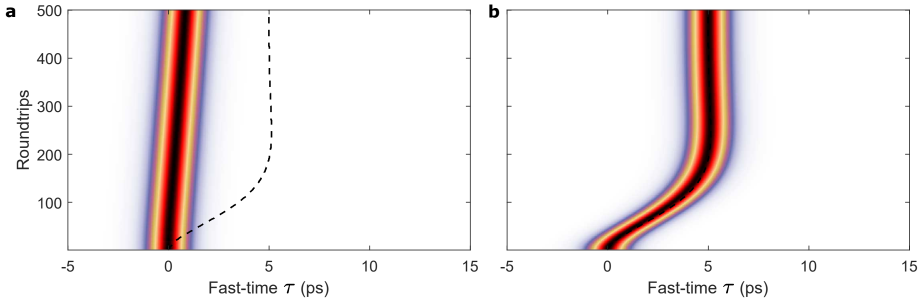

Fig. S3. Internal versus external modulation at $J_{\mathbf{RF}} = 0.05$ rad and $\delta_0 = 0.7$ rad. a, Effect of the external modulation when the potential is suddenly shifted to be centered at a fast-time $\tau_s = 5$ ps. The cavity soliton slowly converges toward the potential minimum. **b**, Same, but with an internal phase-modulation of similar amplitude. Here, the soliton quickly drifts and stabilizes at $\tau_s = 5$ ps. This same situation can be obtained with an external modulation whose strength is augmented by a factor $\mathcal{F}/(2\alpha)$, as illustrated by the dashed line in (a).

F. Supplementary Figures

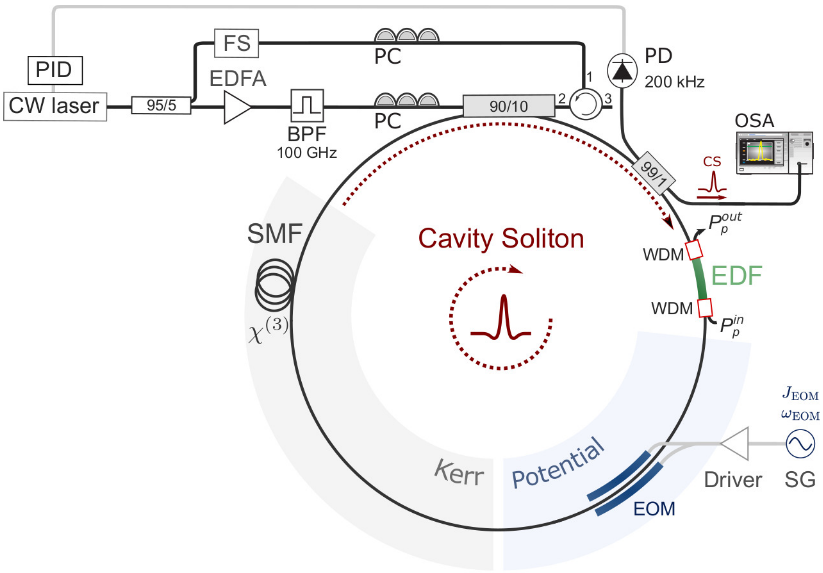

Fig. S4. Experimental set-up. Schematic of the coherently-driven fiber Kerr cavity with intracavity phase modulation. CW laser, continuous-wave laser, FS, frequency shifter, EDFA, optical amplifier, BPF, band-pass filter, PC, polarization controller, EDF, erbium-doped fiber, SMF, standard single-mode fiber, EOM, electro-optic modulator, WDM, wavelength division multiplexer, SG, radio-frequency (RF) signal generator, Driver, RF amplifier, PD, photodiode, OSA, optical spectrum analyzer, PID: stabilization control electronics. We recall that the function of the gain section (EDF) is limited to partially compensating for the loss to emulate a low-loss passive resonator.

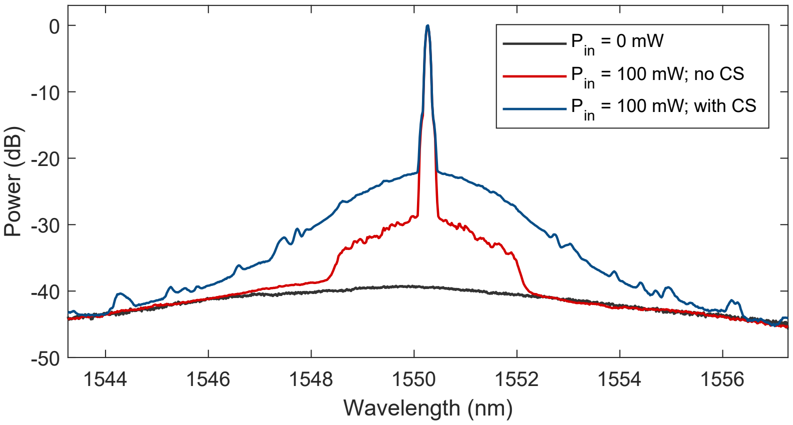

Fig. S5. Spectra recorded at the output of the cavity. Without coherent driving, the spectrum shows only amplified spontaneous emission (ASE) since the active cavity is operated below lasing threshold (black line). With the driving, the spectrum displays a narrowband peak surrounded by a pedestal (red). The latter is a resonant electro-optics comb that originates from the phase modulation of the intracavity driving. A hyperbolic-secant profile is observed once the cavity soliton is excited by the writing pulse (blue). The detuning is $\delta_0 = 2\,\mathrm{rad}$.

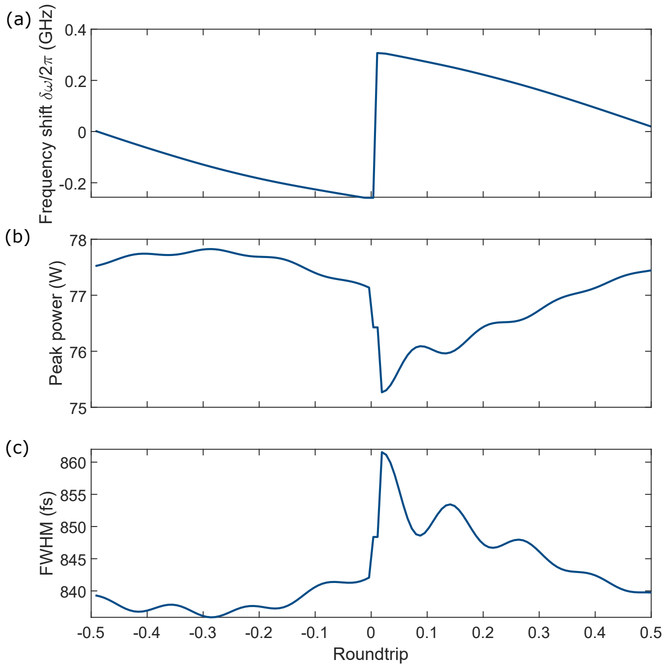

Fig. S6. Soliton single-roundtrip dynamic. Evolution of (**a**) the soliton frequency shift, (**b**) peak power, and (**c**) temporal full width at half maximum (FWHM) over one roundtrip. The simulation corresponds to a lumped element model of the system using the following parameters: $L = 64\,\mathrm{m}$, $\gamma = 1.3 \times 10^{-3}\,\mathrm{W}^{-1}.\mathrm{m}^{-1}$, $\beta_2 = -23 \times 10^{-27}\,\mathrm{s}^2.\mathrm{m}^{-1}$, $\delta_0 = 4.1\,\mathrm{rad}$, $P_{\mathrm{in}} = 250\,\mathrm{mW}$, $\theta_{\mathrm{in}} = 0.1$, $J_{\mathrm{RF}} = 1\,\mathrm{rad}$, $\omega_{\mathrm{RF}}/2\pi = \times 11.8\,\mathrm{rad.GHz}$, $\bar{d} = 0$, $\overline{\tau_{\mathrm{R}}} = 3\,\mathrm{fs}$.

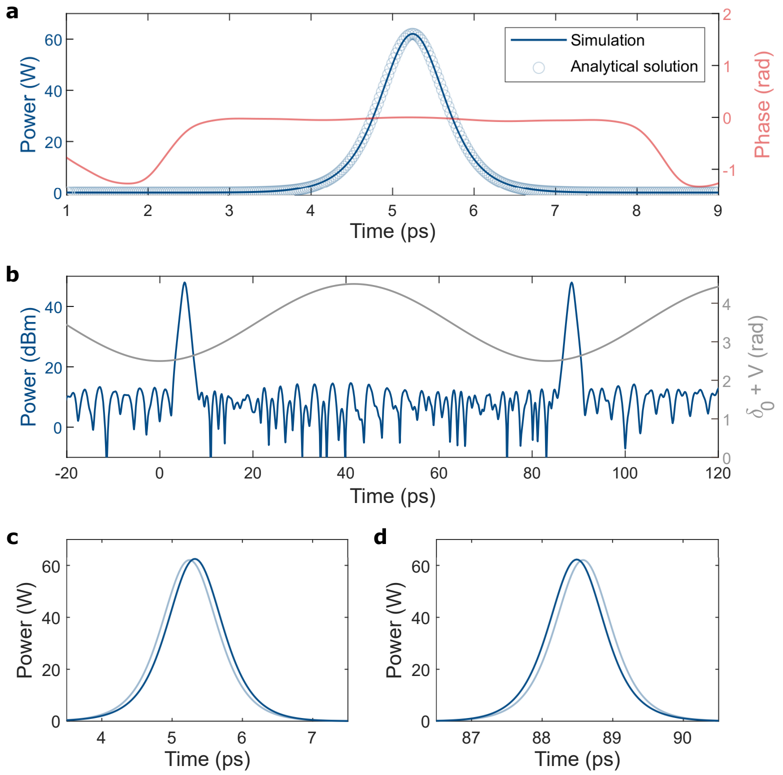

Fig. S7. **a**, Simulation of the cavity soliton with a lump-element model of the cavity including Raman scattering (solid lines). Hyperbolic-secant theoretical profile (circles) whose position in the stationary potential ($d = 0$) is given by Eq.9 of the main paper. The phase of the pulse is nearly flat in agreement with the ansatz in the Lagrangian analysis. We note that the Kelly sidebands cannot be seen in the soliton wings in (a). $\bar{J}_{\mathrm{RF}} = 1\,\mathrm{rad}$, $\nu_{\mathrm{RF}} = 12\,\mathrm{GHz}$, $\theta_{\mathrm{in}} P_{\mathrm{in}} = 10\,\mathrm{mW}$, $\Lambda_{\mathrm{eff}} = 3\%$, $\delta_0 = 3.5\,\mathrm{rad}$. The other parameters are identical to Fig. S6. **b**, Two cavity solitons trapped near two adjacent minima of the potential (shown in grey). The oscillations in the low power background come from the interference with the Kelly sidebands. **(c-d)**, Zoom on the left and the right CSs. The comparison with the simulation of a single CS (light blue) shows that the interaction between the CSs through the oscillating tails slightly shifts the position of the solitons in the periodic potential.

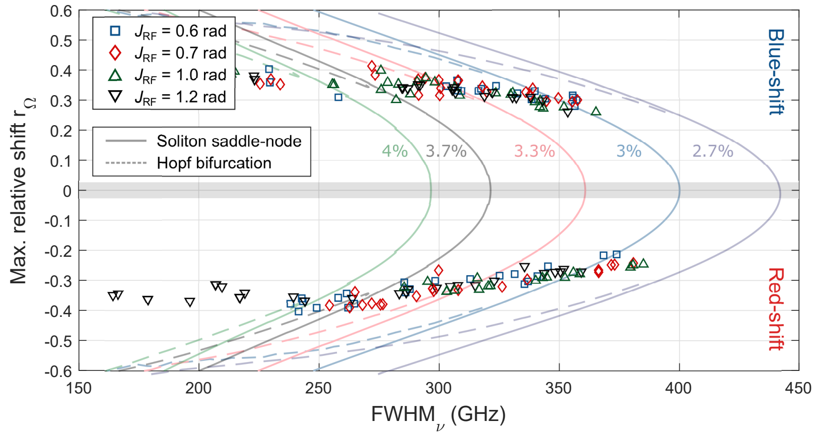

Fig. S8. Experimental maximum relative shift $r_\Omega = \Omega/(2\pi \times \mathrm{FWHM}_\nu)$ as a function of the soliton spectral full width at half maximum (FWHM_ν) for different modulation amplitude J_{RF}. The theoretical region of existence of the cavity soliton, is bounded by the depletion limit (saddle-node) and the Hopf bifurcation. These curves are plotted for different values of the effective loss Λ_{e}.

[1] Luigi Lugiato and René Lefever. Spatial Dissipative Structures in Passive Optical Systems. *Physical Review Letters*, 58(21):2209–2211, May 1987.
[2] Marc Haelterman, Stefano Trillo, and Stefan Wabnitz. Dissipative modulation instability in a nonlinear dispersive ring cavity. *Optics Communications*, 91(5):401–407, August 1992.
[3] Aleksandr K. Tusnin, Alexey M. Tikan, and Tobias J. Kippenberg. Nonlinear states and dynamics in a synthetic frequency dimension. *Physical Review A*, 102(2):023518, August 2020.
[4] Ahmaed Atieh, Piotr Myslinski, Jacek Chrostowski, and Peter Galko. Measuring the Raman time constant (T/sub R/) for soliton pulses in standard single-mode fiber. *Journal of Lightwave Technology*, 17(2):216–221, February 1999.
[5] Xu Yi, Qi-Fan Yang, Ki Youl Yang, and Kerry Vahala. Theory and measurement of the soliton self-frequency shift and efficiency in optical microcavities. *Optics Letters*, 41(15):3419–3422, August 2016.
[6] Nicolas Englebert, Carlos Mas Arabí, Pedro Parra-Rivas, Simon-Pierre Gorza, and François Leo. Temporal solitons in a coherently driven active resonator. *Nature Photonics*, pages 1–6, May 2021.
[7] Andrey B. Matsko and Lute Maleki. On timing jitter of mode locked Kerr frequency combs. *Optics Express*, 21(23):28862–28876, November 2013.
[8] Nicolas Englebert, Nathan Goldman, Miro Erkintalo, Nader Mostaan, Simon-Pierre Gorza, François Leo, and Julien Fatome. Bloch oscillations of coherently driven dissipative solitons in a synthetic dimension. *Nature Physics*, 19(7):1014–1021, July 2023.
[9] Jae K. Jang, Miro Erkintalo, Stéphane Coen, and Stuart G. Murdoch. Temporal tweezing of light through the trapping and manipulation of temporal cavity solitons. *Nature Communications*, 6(1):7370, June 2015.
[10] Jae K. Jang, Miro Erkintalo, Jochen Schröder, Benjamin J. Eggleton, Stuart G. Murdoch, and Stéphane Coen. All-optical buffer based on temporal cavity solitons operating at 10 Gb/s. *Optics Letters*, 41(19):4526–4529, October 2016.
[11] Yadong Wang, Bruno Garbin, François Leo, Stéphane Coen, Miro Erkintalo, and Stuart G. Murdoch. Addressing temporal Kerr cavity solitons with a single pulse of intensity modulation. *Optics Letters*, 43(13):3192–3195, July 2018.
[12] Miro Erkintalo, Stuart G. Murdoch, and Stéphane Coen. Phase and intensity control of dissipative Kerr cavity solitons. *Journal of the Royal Society of New Zealand*, 0(0):1–19, March 2021.
[13] Daniel C. Cole, Jordan R. Stone, Miro Erkintalo, Ki Youl Yang, Xu Yi, Kerry J. Vahala, and Scott B. Papp. Kerr-microresonator solitons from a chirped background. *Optica*, 5(10):1304–1310, October 2018.
